# Desire and Motivation in Predictive Processing: An Ecological-Enactive Perspective

**DOI:** 10.1007/s13164-024-00757-6

**Published:** 2024-12-13

**Authors:** Julian Kiverstein, Mark Miller, Erik Rietveld

**Affiliations:** 1 Department of Psychiatry, Amsterdam University Medical Research, Amsterdam, The Netherlands; 2 Monash Centre for Consciousness and Contemplative Research, Clayton, Australia; 3 Department of Psychology, University of Toronto, Toronto, Canada; 4 Department of Philosophy, University of Twente, Enschede, The Netherlands

## Abstract

The predictive processing theory refers to a family of theories that take the brain and body of an organism to implement a hierarchically organized predictive model of its environment that works in the service of prediction-error minimization. Several philosophers have wondered how belief-like states of prediction account for the conative role desire plays in motivating a person to act. A compelling response to this challenge has begun to take shape that starts from the idea that certain predictions are prioritized in the predictive processing hierarchy. We use the term “first priors” to refer to such predictions. We will argue that agents use first priors to engage in affective sense-making. What has been missing in the literature that seeks to understand desire in terms of predictive processing is a recognition of the role of affective sense-making in motivating action. We go on to describe how affective sense-making can play a role in the context-sensitive shifting assignments of precision to predictions. Precision expectations refer to estimates of the reliability of predictions of the sensory states that are the consequences of acting. Given the role of affect in modulating precision-estimation, we argue that agents will tend to experience their environment through the lens of their desires as a field of inviting affordances. We will show how PP provides a neurocomputational framework that can bridge between first-person phenomenological descriptions of what it is to be a desiring creature, and a third-person, ecological-enactive analysis of desire.

## Introduction

Naturalistic philosophers have tended to work on the assumption that desire is a propositional attitude (see e.g. Dretske 1988; Schroeder 2004). These philosophers take the attitude of desire to be conative with a world-to-mind direction of fit, as contrasted with beliefs that have a mind-to-world direction of fit because they aim at the truth. Standardly it is assumed that there must also be two kinds of learning that map onto the distinction between the attitudes of belief and desire and their different directions of fit (see e.g. Dretske 1988; and in relation to PP, Yon et al. 2020). One type of learning is used by agents to form beliefs whose contents match the world, and to update those beliefs when new evidence is encountered. We will call this ‘epistemic learning’. The other kind of learning allows agents to distinguish the good from the bad, and actions that are worth performing from those that are not. We will call this ‘value learning’. Based on value learning, actions that have preferred outcomes will tend to be repeated, while actions whose outcomes are aversive, or negatively valued, tend to be avoided (Schultz et al. 1997; for philosophical treatments, see Morillo 1990; Schroeder 2004; Railton 2017). Maximizing reward is the primary driver of value learning on the standard account. Value learning consists in learning how to act in such a way as to typically frequent rewarding spaces and avoid punishment (Sutton & Barto 1998).

The predictive processing theory (PP) is an influential family of theories that have in common a view of perception, action, cognition and emotion as working together in the service of prediction-error minimization (Friston & Stephan 2007; Friston 2010, 2011; Clark 2013, 2016; Hohwy 2013; Seth 2015, 2021; Fotopoulou & Tsakiris 2017; Allen & Friston 2018; Kirchhoff 2018; Ramstead et al. 2020; Bruineberg & Rietveld 2014; Bruineberg et al. 2018a; Kiverstein et al. 2019). PP theorists seek to combine epistemic and value learning into a single process of error-driven learning (Friston et al. 2012; Sajid et al. 2021; Smith et al. 2022; Parr et al. 2022). Both epistemic and value learning are conceptualized as the outcome of a single process of prediction-error minimization. Reward and utility is conceptualized as the consequences of action the agent predicts they would sense (through exteroceptive and interoceptive channels), were they to act (Friston et al. 2009, 2010, 2012, 2014; Wiese 2017). Consider as a toy example, my desire for a cup of coffee in the morning. This desire can be modelled as a prediction of a preferred future in which I am now drinking a cup of coffee. I have not yet made coffee, thus prediction errors will arise from my current caffeine-deprived situation. My brain will then predict a course of actions whose future sensory consequences are reliably associated with first making and then drinking coffee. The reward that I derive from my morning coffee can thereby be modelled by predictions, and action control processes that function to minimize prediction errors.

The PP theory of reward and utility has been criticized in the recent philosophical literature on the grounds that it fails to account for motivation (Colombo 2017; Klein 2018, 2022; Yon et al. 2020). Klein (2018) for instance has argued that if all there is to desire is a drive for prediction-error minimization, why wouldn’t a creature, when faced with prediction error, simply change its predictions. Suppose I am starving hungry. Instead of going to the trouble of finding food, I could resolve prediction errors arising from my hunger by updating the prediction that I eat when I am hungry. Of course, this would prove to be a very dangerous strategy in the long run, leading to my untimely death. Klein’s point is simply that updating my predictions seems to always be a viable strategy for creatures whose central imperative is the minimization of prediction error. We follow Colombo (2017) and Klein (2022) in labelling this the *Humean challenge* to PP.^1^

Clark (2019), and more recently Smith and colleagues (2022), have provided compelling responses to the Humean challenge to PP. Both start from the idea that certain predictions are prioritized in the predictive processing hierarchy. Smith and colleagues make appeal to “phenotypic-congruent (preferred) observations that organisms seek out a priori” (p.81). These preferred observations can now be treated as predictions, and deviations from these preferred observations as prediction errors that drive action, as in our earlier coffee example. Clark has likewise argued that predictions that help to keep us alive and viable (p.6) are deeply ingrained and are therefore treated as highly precise. The concept of ‘precision’ refers to an estimate of the reliability of predictions of the sensory states that flow from acting.^2^ Clark recognizes this can only be part of the PP story – not all our intrinsic desires relate to these kinds of biologically installed basic needs. However, he argues that “shifting assignments of precision to varying predictions” (p.7) should do the trick of explaining, more generally, why we are moved by some action possibilities, while other possibilities leave us cold. He suggests his version of the PP theory is no worse-off when it comes to addressing this question than rival accounts that take utility and probability to be represented separately, and independently, in the brain.

In Section 1, we will argue that what both these responses have missed is the role of affectivity in motivating action. The priority that Smith and colleagues (2022) have rightly argued is given to actions that generate phenotypic-congruent observations has the consequence that the body comes to function as a kind of barometer for measuring the affective significance of encounters with the environment. The internal states of the body that must be maintained within a relatively stable range of values if the organism is to survive are examples of phenotypic-congruent observations. Deviation of internal body states from such observations are registered by the body as affective states. The prioritizing of actions that correct for such predictions errors is therefore an example of affect-based regulation of behavior.

We argue (in Section 2) that the predictions prioritized within the processing hierarchy are much broader than those that relate to the maintenance and regulation of the internal states of the body. We borrow the term “first priors” from Allen and Tsakiris (2018) to refer to such prioritized predictions. We will develop an ecological-enactive understanding of first priors according to which predictions that relate more generally to the lifestyle the agent leads in its niche also qualify as first priors.^3^ These predictions underpin an agent’s habits and skills, many of which derive from social and cultural life, and relative to which the agent engages in sense-making. We go on to describe (in Section 3) how affective sense-making based on first priors can play a role in the context-sensitive updating of precision-estimates. We show how, affective sense-making plays a central role in the shifting assignments of precision that Clark (2019) has argued drive action across different contexts. Given the role of affect in modulating precision-estimation, we argue (in Section 4) that agents will tend to experience their environment through the lens of their desires as a field of inviting affordances. We propose to understand desire in terms of a basic concern of the agent to improve grip on the many inviting possibilities that matter to them. The PP account of desire provides a formal, neurocomputational description of the processes that explain why certain affordances are experienced as inviting by a person in a particular context.

### The Role of Affect in Explaining Motivation and Desire

The term ‘affect’ is standardly used to refer to both short-lived episodes of emotion and feeling and longer-lasting persisting moods. In what follows, we do not use the term affect for short-lived and temporary phenomena but for a broader phenomenon of *affectivity* (Colombetti 2014), which we will argue permeates all predictive processing. We will argue that the brain’s predictive processing is organized by what we are calling affective states. We use the term “affect” as it has been defined by Colombetti (2014) to refer to a “lack of indifference…a sensibility or interest for one’s existence” (p.1).

We agree with affective scientists that affectivity has two key features of valence and arousal (see e.g. Russell 1980). To analyze these features using PP it will be useful to introduce Allen and Tsakiris’ (2018) notion of “first priors”—beliefs that relate to the internal conditions of the body that must be regulated and maintained if the person is to continue to exist (see also Fotopoulou & Tsakiris 2017).^4^ First priors, as they propose to understand this class of predictions, can be thought of as specifying a circumscribed set of bodily states that the agent should maintain within a range of values, if they are to continue to exist through time as the individual agent they are. Prediction errors signal a threat to the viability of the body. The actions the organism takes to correct for such threats can therefore be thought of as *autopoietic* (Varela 1979; Maturana & Varela 1980; Thompson 2007) or self-sustaining actions that contribute to the ongoing self-production of the organization of the individual as a complex adaptive living system (Kirchhoff et al. 2018; Kirchhoff 2018; Ramstead et al. 2020; Bruineberg & Rietveld 2014; Bruineberg et al. 2018a). First priors can therefore be thought of as the source of intrinsic biological norms and purposes—the drive for self-maintenance. These intrinsic norms furnish the organism with a kind of inner compass that it can use to evaluate its encounters with the environment in terms of their relevance for its own continued existence.

We propose to characterize this evaluative capacity using the enactive concept of sense-making (Varela 1991, 1997; Thompson 2007; Thompson & Stapleton 2009; Colombetti 2014; Di Paolo et al. 2017). Sense-making entails that the individual has its own point of view or perspective on the environment from which it can bring forth or enact meaning and norms for itself. The encounters of an individual with its environment are sensed in terms of what is good or bad for the self-maintenance of its organization. It is this sensitivity to what is good or bad for the individual that we are calling ‘valence’. Positive valence will tend to move the organism towards aspects of its environment that contribute positively to its self-maintenance. Negative valence, by contrast, will tend to manifest in avoidance behaviors in which the organism seeks withdrawal, escape, or refuses to engage with what is a threat to its self-maintenance.^5^

Bodily arousal, as a second defining dimension of affectivity, can also be connected to motivation and the preparation for action. Arousal has two experiential aspects: (1) tension that is felt along a dimension spanning nervousness to calmness, and (2) energy that is felt along a spectrum from vigor to sleepiness (Wundt 1907, cited by Colombetti & Kuppens 2024). This distinction between different dimensions of felt arousal can be understood physiologically (Colombetti & Harrison 2019). Think here of the role of the sympathetic autonomic system in rapidly preparing the organism to respond to threat either through fight or flight (Cannon 1929): we strike out in anger or run from danger. Activity of the parasympathetic nervous system is by contrast associated with low physiological arousal, and activities of resting and recuperating.^6^ The dimensions of tension and energy can also be understood in terms of sense-making – bodily arousal is what prepares or readies the individual for action. As Frijda (2009) has emphasized, affective states are typically characterized by the organism’s readiness to change or maintain their relationship with an object, event or situation. One need not necessarily act on such a state of readiness – one may refrain from punching a person that has angered one – yet one may nevertheless feel ready to do so. The readiness and bodily preparation to act, felt as a “wanting” or “needing” to act is a part of the lived experience of arousal (Colombetti & Kuppens 2024: p.611).

We propose that affective sense-making can furnish information for estimating precision. Precise predictions are argued to motivate action in PP (see Clark 2019). We will argue that what has not been recognized so far in the discussions of motivation within the literature on PP is the role of affective sense-making in precision-estimation. We will argue that such a recognition opens the door to a more compelling response to the Humean challenge. How does affectivity play a role in precision-estimation? We can provide an initial answer to this question again by reference to what Allen and Tsakiris have called “first priors” (Allen & Tsakiris 2018). Predictions arising from first priors are assigned a privileged role – they are given enhanced precision – within the brain’s processing hierarchy. Consider, for instance, core body temperature in humans that must be maintained within a range of 36.5–37.5°c if the person is to remain alive. The belief that the body will maintain a relatively stable core temperature is an example of a first prior. Allen and Tsakiris’ idea is that because core body temperature is stable and can therefore be predicted with high reliability, if any deviation should be detected it will automatically be conferred a high precision. This precise prediction error will lead to actions that are predicted to return body temperature to its expected value being prioritized over other possible actions. Moreover, prediction errors arising from a deviation from core body temperature will dominate the perceptual processing hierarchy, influencing how the subject perceives the world. The outcome of the privileging of first priors in the predictive processing hierarchy is therefore that the individual will tend to engage in what we have called “self-maintaining” autopoietic actions. These are actions that lead to the sampling of sensory observations that maximize the evidence for first priors – a process Hohwy (2016) has dubbed “self-evidencing”.

Allen and Tsakiris argue that such is the influence of first priors on hierarchical processing that the salience of sensory information can be measured by its positive or negative impact on what they refer to as “visceral and autonomic homeostasis” (p.50). Some first priors concern autonomic functions of the body such as cardiovascular and respiratory control, thermoregulation and glucose metabolism. These are functions that can be registered and regulated autonomically without the involvement of consciousness. Other internal states however relate to bodily needs that are felt with varying degrees of urgency as demanding action from us. Feelings signal that bodily needs are not satisfied that are relevant to the drive for self-maintenance. They inform the organism of deviations from its preferred bodily conditions in which its needs are met. These deviations can be thought of as prediction errors, which because they arise from first priors, are argued by Allen and Tsakiris to automatically be imbued with high precision.

We have used the notion of first priors to explain how affect, via first priors, can play a role in precision estimation. There remain however two key limitations to this line of argument. First priors, as they are characterized by Allen and Tsakiris (2018), are priors that have a phylogenetic origin. The high precision that first priors inherit is restricted in the drive to satisfy basic biological needs. Yet many of our motivations as human agents do not derive from, and may even conflict with, such biological needs. Think for instance of thrill-seeking behaviors in which people are motivated to pursue dangerous, life-threatening activities. Admittedly, such behaviors are not everyone’s cup of tea. Nevertheless, they suffice to illustrate that human motivations are diverse and heterogeneous, extending well beyond those that follow from phylogenetically inherited first priors.

A second limitation is that precision is something that the brain learns to predict and optimize. Precision-weightings on predictions are adjusted by the brain to fit with task and context. Consider for instance how precision weighting might be involved when you search for your keys. In such a context, the brain amplifies sensory information that provides evidence confirming the prediction of finding one’s keys, while at the same time dampening sensory information that is not relevant to this prediction. To accomplish this kind of balancing of sensory information with predictions requires the on-the-fly adjustment of precision-weighting according to context and task. What we have explained so far is how affect could play a role in precision-weighting in the context of what is called “interoceptive inference” (Seth 2013, 2021; also see Barrett & Simmons 2015) in which the task is to keep the internal conditions of the body as stable as possible. We have not yet explained how affect is able to serve such a function in the context and task-dependent weighting of precision in a fast-changing environment. We address these two limitations in the remainder of our paper.

### First Priors: An Ecological-Enactive Interpretation

In the previous section we have seen how autopoietic self-production, through processes of prediction error minimization, can serve as an intrinsic norm or purpose for an organism. This intrinsic norm confers on the individual organism its own point of view or perspective relative to which it can engage in sense-making. The account PP mandates of an individual’s drive for self-maintenance goes well beyond the mere survival of the organism. The highly probable sensory states the individual expects to repeatedly revisit and keep stable reflect the regular patterns of behavior of an individual agent and the ecological niche they inhabit. As Friston has noted, of all the possible sensory states humans could visit, as creatures of habit they tend to repeatedly revisit a small number of sensory states when they get up in the morning, go to work or school, engage in leisure activities and so on (Friston 2019: p.193). What these highly probable sensory states share is that they relate in some way to an individual’s habits and regular everyday activities. They relate to its lifestyle in a niche that, at least in the case of humans, has been historically modified and given shape by social, cultural and material practices (Rietveld & Kiverstein 2014; Bruineberg & Rietveld 2014; Bruineberg et al. 2018b; see also Ramstead et al. 2016; Constant et al. 2018). The identity that is self-produced and sustained by human agents goes beyond the material identity of the agent’s body. The agent’s identity includes its habits such as drinking coffee in the morning. It also includes the skills and abilities the agent develops by growing up in an ecological niche, such as what the agent does in their work life to earn a living for themselves and their family.

The person’s habits and skills will of course change over time. A person can lose a skill, such as for instance speaking a second language, through lack of practice. They can change their habits – for example by quitting drinking coffee. When we talk of the agent’s “identity” we do not mean to claim that the agent exists at each point in time as an identical, single substantial entity across changes in its properties. The notion of identity we have in mind relates to the agent’s organization as an adaptive complex dynamical system that is relatively stable through changes. What it takes for the agent to continue to exist as an adaptive complex system is for the system’s organization to be continuously self-produced and maintained. We are arguing that the individual’s habits and skills are among the processes that are self-produced and maintained, contributing to the individual’s identity. You are what you do, and conversely, you do what you do in your life because of who you are (Di Paolo et al. 2017: p.142; cf. Bergson 1922: 7). The ecological-enactive interpretation of first priors we are proposing takes the category of first priors to include habits and skills developed over the individual’s lifetime. Just as the body’s integrity is self-produced and maintained, so also are the habits and skills that constitute the individual’s existence.

PP entails that agents that aim to keep prediction errors to a minimum over time will tend to act to minimize the divergence between the future situation they predict – one in which the bodily conditions of their existence in all their complexity are sustained, and the future they experience. This is equivalent to saying that they will tend to select sequences of actions (i.e. action policies) that minimize *expected* (i.e. future) prediction errors (Smith et al. 2022). This requires forecasting the future consequences of their action policies – the changes in hidden states over time that would ensue were each action policy to be selected. Knowing how policies influence transitions in sensory states over time allows the agent to compute the likelihood of sensory observations under each policy. This likelihood can then be combined to compute the posterior probability of pursuing each policy, where policies are each given a score based on their associated expected prediction errors. By selecting policies with the lowest expected prediction errors, the agent is thereby making a prediction that it is highly likely they will follow a path that leads from their current situation to a future they prefer in which their conditions of bodily existence are produced and maintained.

Smith and colleagues (2022) make the important observation that the expected prediction error associated with a given policy doesn’t only provide a score of how likely the selection of a policy is. It can also be thought of as, in their words, “encoding the overall “drive” to choose one course of action over another” (p.80). The motivational power of desire can thus be modelled as the divergence between the agent’s current situation in which some precisely predicted aspect of its bodily existence does not yet obtain, and the future situation it expects to bring about through its actions in which these conditions are realized. There is no inconsistency between the account of motivation we have been developing and that of Smith and colleagues. Expected prediction errors are a function of two quantities – risk and ambiguity. Risk refers to the divergence between expected and preferred outcomes. Preferred outcomes are predicted based on what Smith and colleagues call the “prior preference distribution” which they define as encoding observations congruent with the organism’s phenotype. Smith and colleagues’ prior preference distribution is what we have been interpreting in terms of first priors understood in ecological-enactive terms. What our account adds to Smith and colleagues’ treatment of desire and motivation is therefore an explicit recognition of the role of affectivity as what motivates action in PP. Affective sense-making is arguably implicit in their account, hidden behind their talk of preferred, and phenotypic-congruent observations. We bring affectivity into the foreground.

### Precision Engineering

Let us turn now to the second limitation of the PP account of motivation we have proposed so far in this paper. Recall that this was the question of how precision estimates are adjusted and optimized in ways that are task and context dependent. Modulating the precision of an error signal requires predicting precision across different contexts and calculating deviations from predictions that can be used to up or down-weight precision estimates. We tend to trust our eyes for instance in day light under conditions of good visibility, while at night-time, in the dark, we may rely more on our ears or on touch. PP will need to include an account of how the brain adjusts its assessments of the reliability of its predictions. This is to say that PP must include in its model of the brain, a capacity for estimating expected uncertainty that can be used to evaluate how confident the brain should be in its predictions in its current context.

Solms (2021) has proposed that the function of keeping track of expected uncertainty is one that physiological arousal can play (also see Solms & Friston 2018; Solms 2019). Physiological arousal is, he has proposed, a response to “fluctuations in expected uncertainty” (p.203). By making interoceptive inferences about changes in physiological arousal, such as change in heartrate for example, the brain can thereby keep track of changes in expected uncertainty (Allen et al. 2016; Biddell et al. 2024). Prediction errors arising from unanticipated changes in physiological arousal, we propose to understand in enactive terms of sense-making. They are interpreted by the individual against a background of the drive for self-maintenance. Thus, what unanticipated changes in arousal signal for the individual is potentially significant risk to their own viability that ought to be corrected for. These prediction errors can thereby be used to adjust upwards or downwards expectations of uncertainty and thus to modulate precision-weightings according to context.

Valence has also been modelled in PP as playing a role in context-sensitive updating of precision estimates. Hesp and colleagues for instance describe a model of valence as tracking moment to moment changes in what they have called “subjective model fitness”—confidence in the model that computes the expected prediction errors used to select between action policies. Hesp and colleagues introduce the notion of ‘affective charge’ – a state that tracks how well or badly an action model – a model of a sequence of actions and their sensory outcomes – performs over time at error reduction.^7^ Error reduction refers to the divergence between the future the agent expects when it acts – one in which its conditions of bodily existence are realized – and the future the agent experiences. Decreased in confidence in a model is argued to lead to inference of negative feelings, signaling that the agent should not rely on the model but instead fall back on past behaviors and habits. In contexts in which confidence in an action model is low, the agent should rely on evidence from the past in the form of the habits encoded in the action model, not on expected prediction errors to select actions. Increase in confidence in a model is argued to form the basis for inference of positive feelings. It signals that the agent is confident that a model of action policies and their outcomes can be relied upon to bring about preferred sensory outcomes. By contrast when confidence in the action model is high, an agent agent should select policies that minimize expected prediction errors. Affective charge can therefore be used to regulate whether the agent relies on its history of activity or on projections of the future in selecting what we have called autopoietic, self-maintaining actions.

Let us briefly pause to connect the strands of our argument. In Section 1 we have argued that valence and arousal, as dimensions of affect, should be interpreted as arising from divergences from first priors that reflect a norm of self-maintenance. Section 2 argued for expanding the reach of first priors beyond the organism’s internal bodily states to its wider bodily existence in the world. First priors should be understood in the broader context of habits, skills and abilities an individual has developed and embodied over the course of their life. Affect is modelled by prediction errors that signal deviation from first priors understood in ecological-enactive terms. Now consider the hypotheses we have outlined in this section that arousal and valence play a role in the ongoing context-sensitive modulation of precision-weighting. This involvement of arousal and valence in the context-sensitive adjustment of precision-weighting will ensure that the prediction errors that are prioritized by the individual’s nervous system are relevant in some way to the realization of the individual’s bodily conditions of existence. First priors are a measure of what is salient and what is not. Salient sensory information provides evidence for currently prioritized predictions, the predictions that flow from first priors. It follows that all sensory information (interoceptive and exteroceptive) will be processed in terms of its bearing on, or implications for an individual’s self-production and maintenance of their identity as an agent acting in a meaningful environment.

Another way to put this is to say that the individual will select (i.e. ‘enact’ or ‘bring forth’) its own meaningful world. In the enactive literature, sense-making is described as an individual’s capacity for “enacting” or “bringing-forth” a meaningful environment. As Thompson and Stapleton (2009) put it, sense-making is what transforms a meaningless physical reality into a “place of salience, meaning and value, an environment (*Umwelt*) in the proper biological sense of the term” (p.25). We will describe in the next section how the *Umwelt* for an individual can be described in the terms of ecological psychology as a field of inviting affordances (Withagen 2022, 2023; Bruineberg & Rietveld 2014; Rietveld & Kiverstein 2014; Rietveld et al. 2018). Thus, the predictive processing theory of motivation we have been outlining can be thought of as an explanation of why certain affordances stand out as inviting to an individual perceiver. It is inviting affordances that motivate action.

### Being Moved by the Possibilities that Matters to Us

A creature that is moved through the world by affect in the way we have been describing will experience an environment of inviting affordances. Given the priority that is given to first priors in the predictive hierarchy, the world will typically show up for them as a field of inviting affordances. We use the term ‘affordance’ to refer to a possibility for action offered by the environment, such as the catchability of a mouse for a cat (Gibson 1979). We use the term ‘inviting affordance’ to refer to the affective significance an affordance has for an organism (Dreyfus & Kelly 2007; Withagen 2022, 2023; Rietveld & Kiverstein 2014; Bruineberg & Rietveld 2014; Rietveld et al. 2018). Inviting affordances have a positive or negative valence. They attract or repel, making demands on us with a certain force or intensity. In acting we respond to this demand; it is as if our movements are drawn from us by the affordances we encounter as inviting (Dreyfus & Kelly 2007). In taking a step back from someone that has just entered an elevator with us, for instance, one is literally moved to take up a certain distance. If we fail to take a step back and the person stands too close to us, this may feel uncomfortable, and it is this anticipated feeling of discomfort that moves us to act.^8^

It is inviting affordances that account for what an agent is motivated to do. This fits with phenomenological accounts of skilled action that describe how skillful agents experience their actions being drawn from them by the situation in which they find themselves (Dreyfus & Kelly 2007). Think for instance of the classic example of the speed chess player that sees immediately which moves to make as a game of chess unfolds. We propose that it is affect, and its role in the modulation of precision-weighting, that can be used in PP to model why certain affordances are inviting in a particular action context. Agents will tend to select actions they believe will realize their conditions of existence.

Smith and colleagues (2022) have described the formal details of how the objects of desire can be modelled as prior preferences. The argue that representations of desired outcomes can be mapped onto prior preferences. A desire for ice cream for instance is, on their account, explained by the discrepancy between the agent’s current situation and the future they prefer in which they are eating ice cream (just like in the coffee example with which we started our paper). We have just suggested the PP account of desire provides a formal description of why particular affordances invite behavior in a particular situation. Does it follow that the question of why some affordances invite behavior can be explained by reference to an agent’s prior preferences (i.e. by representations of desired outcomes)? We do not think so. We will use this question to identify some differences in the ecological-enactive perspective on PP we have opened up through our discussion of affective sense-making and its role in precision-weighting, and Smith and colleagues work.

First, a point of agreement between us and Smith and colleagues. We agree that desires can be modelled as arising from the mismatch between the agent’s current situation in which there is always some preferred conditions of existence that do not obtain, and the future situation which the agent prefers, and confidently believes it is able to bring about through acting.^9^ However, we will argue that affordances do not invite behavior because of a desire for this or that particular object or outcome.

In our ecological-enactive account, affect can be thought of as arising from a state of disequilibrium in the system the agent forms with its environment from moment to moment. The state of disequilibrium provides the organism with affective feedback about how well it is faring in its engagement with the world. Here we take our inspiration from Merleau-Ponty’s later writings on the philosophy of nature where he describes a disequilibrium, an inherent instability, within the organism that is the driver, the motor, of the organism’s self-movement through its environment (Merleau-Ponty 2003).^10^ In an attempt to correct for this instability, the organism is drawn into action by those affordances it anticipates will move it closer towards a state of relative equilibrium. Elsewhere we have described, in phenomenological terms, how the affordances that invite action are experienced by a person as improving their grip in a particular situation of action (Bruineberg & Rietveld 2014; Bruineberg et al. 2018a; Rietveld et al. 2018; Kiverstein et al. 2019). The tendency towards an improved grip refers to a phenomenological structure of lived experience that was first described in Merleau-Ponty (2011). Think of how in an art gallery we stand at the right distance from a painting to take in its details, or when we are looking at a specimen through a microscope, we turn the dial on the microscope to gain a better view of the specimen. What we are doing in these examples is moving to improve grip upon the world. Grip refers to a bodily stance an agent must actively maintain in relation to its current situation. We talk of grip as tending towards *improvement* because the skilled agent is always sensitive to doing better or worse, adequately or inadequately, appropriately or inappropriately in a particular situation, and is moved to improve their grip on the situation.

We propose to think of the tendency to improve grip as a basic concern, intrinsic to being alive, that animates an agent’s interactions with its environment. We borrow the term “concern” from the emotion psychologist Nico Frijda. He took an organism’s concerns to refer to “what gives events their emotional meaning” (Frijda 2007: p.123). The function of emotions in general, according to Frijda, is to safeguard a person’s concerns. The tendency to improve grip can be thought of as a basic concern in the sense that it forms a phenomenological structure common to all the other emotional concerns of the organism. We suggest that the PP account of motivation in terms of affect-based precision estimation provides formal tools for computationally modelling this phenomenological structure of tending towards an improved grip. Phenomenologically, it is tending towards an improve grip that describes our experience of being invited to act in a particular way. PP provides a formal model of this structure of lived experience by reference to first priors and the affectivity that follows from their temporary violation that the agent is then moved to correct.

We can think of the process of action selection in terms of the formation of complex states of action readiness that anticipate the sensory consequences of an organism’s current and future practical activities in the environment (Bruineberg & Rietveld 2014; Bruineberg et al. 2018a; Rietveld et al. 2018; cf. Anderson 2017; Gallagher 2017; Allen & Friston 2018). The organism’s states of action readiness are predictive in the sense of potentiating or preparing the agent to act in relation to the world. Each state of action-readiness forms as an expression of an individual’s affective sense-making that registers how the individual is faring in its current situation. Consider a cat that readies itself to pounce on a mouse it spots out of the corner of its eye. The cat’s readiness to pounce is a structured whole, comprising processes of neural potentiation, accompanying changes in physiology and musculature, autonomic responses such as modulation of heart rate, and hormonal changes such as the production of adrenaline, that self-organize because of a sensory perturbation—the fleeting sight of the mouse moving into its field of view. Crucially, at the same time as the cat is coordinating its states of action readiness with the mouse, it is also ready for the neighbor’s dog, the chair on which it likes to sleep, its owner, and many other possibilities besides.^11^ This coordination happens over multiple time scales. Thus, right now the cat is engaged with the mouse, but the cat may also be simultaneously ready to laze in the sun once the mouse has been captured.

We have suggested that motivation is best thought of in relation to inviting affordances that move us to act because they allow for us to improve grip on what matters to us. Why not understand the objects of desire instead in the way Smith and colleagues (2022) have proposed in terms of representations of preferred sensory outcomes? The short answer to this question is that our account follows from the role we have assigned to affective sense-making in motivating action. As already noted briefly above, such a role for affect is arguably implicit in the account of Smith and colleagues since what they call prior preferences map onto what Allen and Tsakiris call “first priors”. We would also make a stronger claim. It is necessary for affectivity to play the role we have assigned to it if predictive processing is to account for motivation. Consider again the way in which Smith and colleagues use expected prediction errors to account for motivational drives. They suggest that it is the magnitude or size of the prediction error between my current situation in which I am not eating ice cream and the future in which I am eating ice cream that accounts for why I am motivated to seek ice cream. They describe the difference between three agents – one who strongly desires ice cream, a second who weakly desires ice cream and a third who has no desire whatsoever in terms of the magnitude of the magnitude of prediction errors arising from the three agent's prior preferences. The first agent will have a larger prediction error than the other two agents, which they take to explain the urgency with which the first agent seeks ice cream. Why does the size of the prediction error make a motivational difference? Why couldn’t an agent token such a large prediction error and not be motivated to take any action of seeking ice cream? It seems that what makes the motivational difference is that eating ice cream is something that matters to different degrees to the three agents.^12^ Where it not the case that eating ice cream affects them in some way, we suggest it would not count as a strongly preferred outcome.

Now consider Clark’s account of motivation in terms of shifting assignments of precision. Clark writes: “As both our inner states (hunger, thirst, etc.) and outer contexts ebb and flow, some predictions enjoy increased precision, becoming positioned to drive immediate action, while others remain in the background, awaiting the right opportunity to arise” (p.7). He also does not explicitly make mention of the role of affect in motivating action, although we note that elsewhere Clark has emphasized that prediction errors are calculated by the brain relative to “affectively rich and personal-history-laden expectations” (Clark 2013: p.200). We can ask the same question of Clark that we just asked of Smith and colleagues – why is it that the predictions that enjoy increased precision are the ones that motivate action? Could a prediction of sensory outcomes not enjoy increased precision, promoting this prediction over other predictions but not move the agent at all? We suggest again that what makes the difference is affect – the predictions that are prioritized are typically evaluated for priority in a hierarchy in which first priors are always privileged. We have argued that affective states arise because of prediction errors that relate to first priors.

It might be objected that Clark and Smith and colleagues could agree with us about the role of affect in precision-estimation and in motivating action, while continuing to think of desires as mapping onto representations of preferred sensory outcomes. In other words, they need not agree with the phenomenological description we have given of motivation in terms of improving grip through being drawn into action by inviting affordances. In their paper, Smith and colleagues set about showing how desires at a psychological level of description can be “straightforwardly identified” with Bayesian beliefs at the mathematical level of description (p.81). They argue that “Bayesian beliefs play the same functional role as representations of desired outcomes” (*Ibid*.), and they note that their account is broadly consistent with that of Clark (2019). Clark argues that PP has replaced the traditional constructs of “beliefs, desires and rewards with a single construct – that of predictions, which when they are held with high precision…entrain apt action” (p.7). The probabilistic priors that sculpt predictions are, Clark argues, both belief-like and desire-like (p.12). What is common to both these accounts is that they take the explanatory target to be beliefs and desires understood in folk psychological terms.

The explanatory target of our account has turned out to be somewhat different. A key motivation for the ecological-enactive project is to expand the horizons of cognitive science to find a place for lived human experience described phenomenologically in terms of the mutual dependence and co-origination of body and world (Varela et al. 1991). A central idea in this work has been that body and world co-arise together and therefore stand in a relation of mutual dependence. We have seen an example of this in the description we have given of how inviting affordances on the side of the environment, and states of action-readiness on the side of the body, form together based on a basic concern to improve grip on their environment.

As will be obvious by now, we share the ambition of the ecological-enactive project in cognitive science to find a place for lived experience within cognitive science. We have presented a perspective on PP as providing computational formal modelling tools that allow for “passage” (Ramstead et al. 2022; Varela 1997) between first-person phenomenological descriptions of desire, and the physiological and somatic processes that desires depend upon as described in third-person terms. The mathematics of computational modelling provides a language that is neutral between desire as a phenomenological invariant structure of lived experience, and invariants in the dynamics of the body in its coupling with the world.^13^ Our aim has been to show how PP provides formal tools that can be put to work to map the structure of lived experience onto agent-environment dynamics as studied in ecological-enactive cognitive science.

Should we conclude then that the ecological-enactive account of desire we have developed on the basis of PP is targeting a concept of desire distinct from that found in the earlier work on PP?^14^ A possible way to relate our two accounts is to see PP as providing formal tools that allow for passage between first-person phenomenological descriptions of what it is to be a desiring creature and third-person psychological explanations that take conative states of desire (in conjunction with beliefs) to causally explain a person’s behavior. Previous work has been concerned with showing how the states appealed to in psychological explanation can be identified with the mathematically defined states appealed to in the models constructed using the tools of PP. We have argued that PP can be used to formally model a phenomenological structure of lived experience – the tendency towards an improved grip on affordances. One option would therefore be to view our paper as arguing that PP provides formal, mathematical tools for building a bridge between first-person phenomenological description and third-person psychological and neurocomputational explanations of behavior.

However, whether our ecological-enactive perspective is compatible with the earlier work in PP on desire turns on a larger issue we have been skirting so far in our article about how to make sense of the intentionality of desire in naturalistic terms. Smith and colleagues and Clark both endorse a representational understanding of mind (see also Kiefer & Hohwy 2018 and Kiefer 2017). Proponents of the ecological-enactive theory have, by contrast, typically argued for a *non-representational* understanding of the mind on the grounds that body and world stand in a relation of mutual dependence. Ecological-enactive theorists have followed phenomenological thinkers in arguing that lived experience is what makes possible the cognition of objects, which is where we typically find representational modes of thinking at work. Ecological-enactive theorists would therefore challenge the project of naturalistically explaining representation in terms of inferential processes inside of the brain because they take representational modes of thinking to have their origins not in the brain, but in lived experience.

In this section, we argued that PP offers formal tools for modelling the phenomenology of desire. We have described desire in phenomenological terms as the tendency to improve grip on multiple inviting affordances that matter to the agent. PP can be used to formally model this phenomenological structure. Thus, PP can contribute to a project of naturalizing the intentionality of desire states without necessarily endorsing the representational theory of mind.

## Conclusion

Various philosophers have argued that prediction is powerless to explain the motivational force of desire (Klein 2018, 2022; Colombo 2017). Critics of PP have argued there is a gap between prediction and action that can only be bridged if conative states are something other than predictions. Both Clark (2019) and Smith et al. (2022) have responded to this Humean challenge to PP by arguing that precise predictions have the right functional profile to motivate action (Clark 2019). In PP, desired outcomes are modelled as high-precision predictions. (Clark 2019; Smith et al. 2022). We have argued that while correct, what these accounts have so far failed to make explicit is the role of affect in motivating action. We have argued that affect can play this role because it arises out of violations of what we have called “first priors” (a term we borrow from Allen & Tsakiris (2018)). Predictions that follow from first priors are given enhanced precision in the processing hierarchy. First priors, insofar as they generate self-maintaining actions, we have argued furnish the individual with an affective point of view on its environments. Given the place that first priors occupy in the processing hierarchy, all sensory information is evaluated in terms of it bearing on the ongoing self-production of the organization of the individual as a complex adaptive living system (Bruineberg et al. 2018a; Kirchhoff et al. 2018; Kirchhoff 2018; Ramstead et al. 2020).

We have proposed an account of first priors in ecological-enactive terms as relating to the agent’s bodily existence in the world. We described how affective sense-making, based on first priors, can play a role in the context-sensitive updating of precision-estimates. We have argued that the resulting account of desire has implications for how the subject experiences their surrounding environment. It follows from the priority that is given to first priors that desire will structure the affordances the subject experiences as inviting them to act.

We finished up considering points of difference between the project we have been engaged in of providing tools for modelling lived experience and earlier work that was our point of departure in this paper. The earlier work has focused on showing that PP has explanatory resources for making sense of conative states. Our project, by contrast, has ended up targeting lived experience. We have argued, based on the PP account of desire, that subjects should experience their surrounding environment through the lens of their desires. What they experience can be described as a field of inviting affordances. We have used the PP resources for modelling desire to answer the question of why some affordances but not others invite an individual to act. Desire can be described in phenomenological terms as a basic concern to improve grip on a field of multiple inviting affordances. We have argued that PP provides computational tools for modelling the phenomenological structure of tending towards an improved grip on the environment. Thus, we have used the formal computational tools of PP to provide passage between first-person, phenomenological descriptions of lived experience of being a desiring creature, and psychological explanations of desire and motivation as a cause of our behavior.

## Data Availability

There are no experimental subjects and no data available.

## Footnotes

1 The challenge builds on Hume’s argument that beliefs and desires have a different causal profile, and beliefs, taken on their own, have no motivational force (Hume 1978; see Smith 1994 for discussion). Predictions and error reduction seem to have the folk psychological profile of beliefs and belief update. Thus, the machinery of prediction and prediction-error minimization looks like it is not well-suited to modelling desire and motivation.

2 Precision is defined more formally as the inverse of variance in the data (i.e. the sensory outcomes of an action) around the mean of a probability distribution. The more variance the less reliable or precise the prediction is. The predictions that get to drive action are therefore those that are estimated to have the highest precision. So long as the precision estimates are accurate and reliable, the ensuing actions should be a means to fulfilling the agent’s predictions.

3 In the recent literature points of tension have been identified between PP and the ecological-enactive framework in cognitive science (see e.g. Di Paolo et al. 2022). We agree with Di Paolo and colleagues that the mathematical tools of PP can, and have been given interpretations, that conflict with the core tenets of the ecological-enactive approach. However, we will argue (in Sect. 4) that PP is perhaps best interpreted as providing formal tools for computationally modelling lived experience. Thus, PP can complement the aims of ecological-enactive cognitive science to enlarge the horizons of cognitive science to find a place for lived human experience within a scientific model of the human mind. It is beyond the scope of this paper to provide a response to Di Paolo and colleagues more specific arguments about the incompatibility of their enactive approach and PP but for some starting points towards addressing their challenge, see Kiverstein et al. 2022.

4 Note that whenever we use the notion of “prior belief” in this paper we do not mean to refer to a folk psychological state of belief. We use belief to refer to a state in a computational model that encodes a probability distribution. A first prior, as Allen and Tsakiris use this concept, refers to a probability distribution in a model whose parameters relate to internal states of the body, where the distribution is sharply peaked around what are called homeostatic setpoints.

5 As Colombetti & Kuppens (2024) have argued, some caution is needed here in aligning the distinction between positive and negative valence with approach and avoid behaviors. Take for instance anger – often a negative emotion – in which the person may be motivated to attack (i.e. approach) the target of their anger. Colombetti & Kuppens also rightly highlight the potential for confusion in the multiple ways in which the term “valence” is used in the psychology literature. Valence is used in affective science to refer to experienced feelings, behaviors and cognitive states of evaluation and appraisal.

6 Colombetti & Harrison (2019) argue that physiological arousal is not exhausted by the response of the autonomic nervous system. Also playing an important role in underlying arousal are the gastrointestinal, immune and endocrine systems. The stress response for instance is characterized by the release of hormones from various endocrine glands that dynamically interact with brain activity.

7 We should note that we have changed the wording of Hesp et al. (2021) here to fit with our terminology. They define affective charge as tracking changes in estimates of subjective fitness (p.399), and they define “subjective fitness” as the “perceived efficacy of an organism’s action model in realizing phenotypic-congruent (i.e. preferred) outcomes” (p.402). An anonymous reviewer objected that Hesp and colleagues’ treatment of affective charge entails a representationalist interpretation of PP. They suggest that the perceived efficacy of an action model requires that the organism take a model to be performing well or badly at bringing about phenotypic-congruent outcomes. We turn to our reasons for rejecting such a representational reading of PP below, which derive from our commitment to an ecological-enactive conception of mind. For now, we note that the implicit non-reportable metacognition (p.408) that Hesp and colleagues take to be involved in tracking subjective fitness need not be thought of as involving representational content. We define “content” in terms of states that take (e.g. claim, say, assert, picture) things to be a certain way, such that things need not be the way they are taken to be (Hutto & Myin 2012). In other word, content is carried by states that can be mistaken, inaccurate, incorrect, or false in the way they take things to be. We do not agree with the reviewer that implicit metacognition needs to be interpreted in content-involving term since it is a type of metacognition that is non-reportable. Content in our view is semantic, characterizable in terms of truth and reference, and it is only propositions that carry semantic content.

8 It should be unsurprising that perceptual experience includes such an affective dimension given what has been argued so far. In PP perceptual experiences are the outcome of cascades of prediction that enfold affect and value because of the role of first priors in privileging certain predictions in the processing hierarchy.

9 Do situations in which we desire for our current experiences to continue count as counterexamples to this proposal? Suppose we are fortunate to have a job we enjoy in the present and would like that we hold onto this job in the future. In such a situation we have what we want, and our preferred future is one that matches our current situation. Still to bring about a preferred future requires action. The future has not yet happened. Thus, from our vantage point in the present, there is a mismatch between our current situation in which we are doing the job we enjoy and the future we would prefer to inhabit in which this continues to be the case, a gap that we attempt to close by taking the necessary actions. Moreover, even if we suppose that an individual’s work life is going well, the individual will typically have many other cares and concerns that make for divergences between their current situation and the future they prefer which they are motivated to act to reduce.

10 There is an interesting thread connecting Merleau-Ponty’s later writings on life to our earlier discussion of the experience of inviting affordances, which we unpack further below (though without reference to Merleau-Ponty).

11 Pezzulo & Cisek (2016) have modelled intentional action “as a purposive navigation in an ‘affordance landscape’: a temporally extended space of possible affordances, which changes over time due to events in the environment but also – importantly – due to the agent’s own actions” (p.415, see also Pezzulo et al. 2018). Their model of intentional account bears a striking resemblance to our proposal to understand organisms as simultaneously poised over a field of multiple relevant affordances. There are however important differences between what we call the field of relevant affordances and what Pezzulo and Cisek call “the affordance landscape”. The most important difference is that we characterize action relationally in terms of readiness for multiple relevant affordances. The cat for instance, is simultaneously ready for the mouse, its owner, the neighbor’s dog and so on. Pezzulo and Cisek by contrast characterize intentional action in neurophysiological terms of competition between multiple neural representations of affordances. In the end, there is one affordance as represented in the brain that wins the neural competition and gets to drive intentional action. This contrasts with our suggestion that skilled agents embody complex states of action readiness that prepare them to act on multiple relevant affordances simultaneously (see Bruineberg & Rietveld 2014; Rietveld et al.
2018 for an elaboration of this claim and discussion of relevant empirical evidence).

12 It should be noted that Smith et al. (2022) do not claim that the size of prediction errors is sufficient to account for motivation. They would agree with the account we have been developing in this paper of the privileged role of first priors in the predictive processing hierarchy. This is implicit in Sect. 1 of their paper in which they explain how the predictions over observations in the generative density derive from phenotypic priors. (Thanks to an anonymous reviewer for emphasizing this point to us.) Our disagreement with Smith and colleagues, as we go on to explain, concerns how to account for the intentionality of desire, and whether preferred outcomes as the intentional objects of desire should be viewed as represented by the brain. We are proposing to view preferred outcomes in non-representational, relational, environment-involving terms. More specifically, we propose to understand preferred outcomes as inviting affordances that the whole embodied organism tends towards an improved grip on.

13 Kiefer (2020) offers an interpretation of PP as supporting a psychophysical identity theory. The mathematical model can be described cognitively in terms of contents of computational states or physically “in terms of the energy dynamics governing the (neuronal vehicles) of its representations” (p.2). We have suggested above that the model is neutral between a phenomenological and a physical description. Where we depart from Kiefer is in questioning whether the physical description is representational for reasons we explain above.

14 One could argue that there is no opposition between the account of desire we propose and that of Smith and colleagues and Clark because we are simply asking different questions. We are concerned to explain inviting affordances while Smith and colleagues and Clark are concerned with analysing the functional role of desire using the formal tools of active inference. We arrive at different, but compatible, understandings of desire because we have different questions we are looking to PP to answer. Whether such a response works depends on larger questions about the representational theory of mind challenged by proponents of the ecological-enactive view of the mind. Some philosophers have recently argued for a reconciliation of enactive and representational theories of mind using active inference (Constant et al. 2021). Engaging with these arguments is however beyond the scope of this article.
